# Supplementary material for: ATAC-Seq analysis reveals a widespread decrease of chromatin accessibility in age-related macular degeneration
Source: Nat Commun. 2018 Apr 10;9:1364. doi: 10.1038/s41467-018-03856-y (PMC5893535; doi:10.1038/s41467-018-03856-y)
Supplement: Supplementary file 1 — Supplementary Information [file 41467_2018_3856_MOESM1_ESM.pdf]

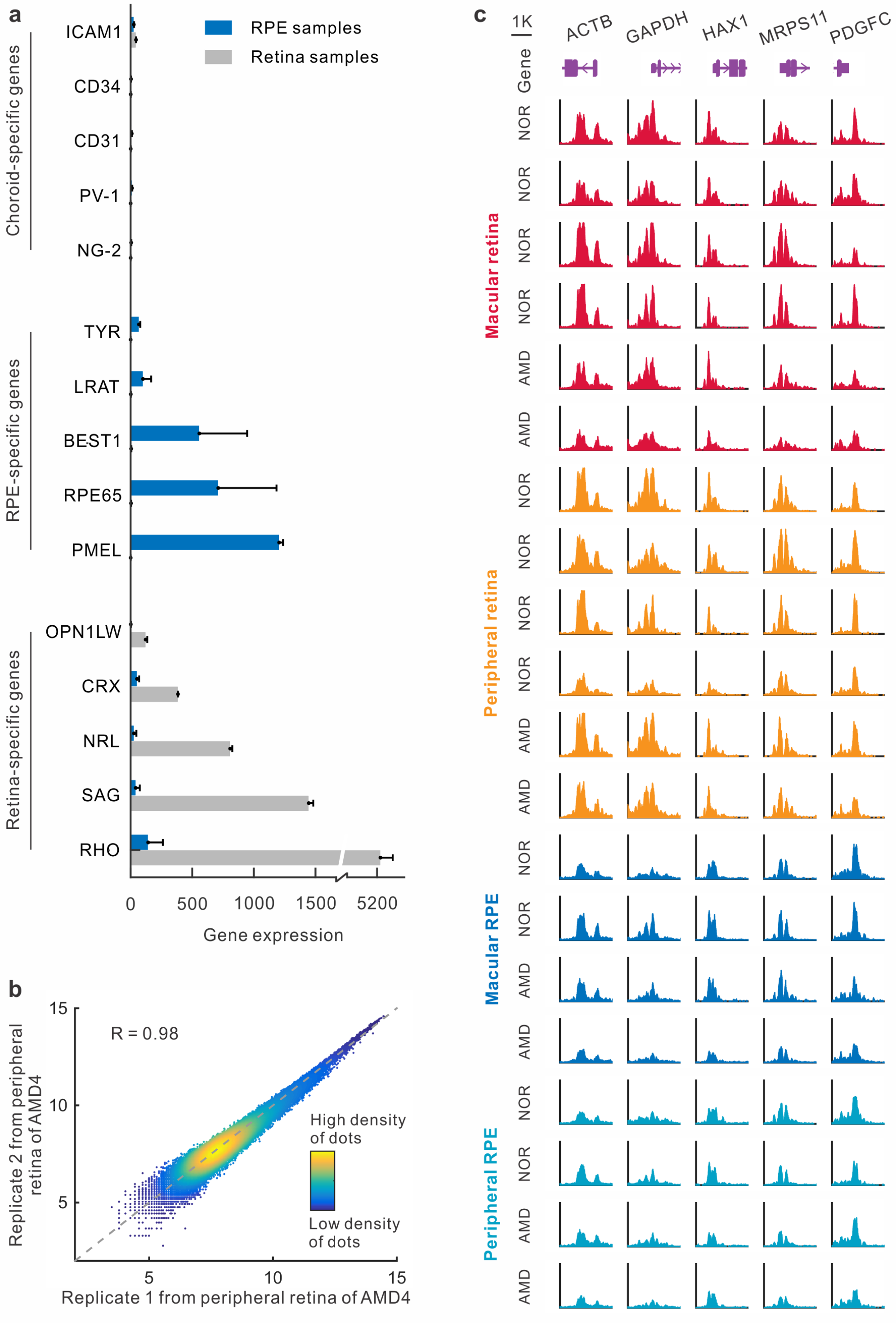

Supplementary Figure 1. Quality of retinal and RPE samples, and ATAC-Seq reproducibility. **(a)** Expression of tissue-specific genes in retina and RPE samples (n =2). The error-bar represents the standard error of the mean. **(b)** ATAC-Seq comparison of the replicated samples from adjacent regions in peripheral retina of right eye from one AMD patient. **(c)** Examples of ATAC-Seq peaks associated with typical housekeeping genes.

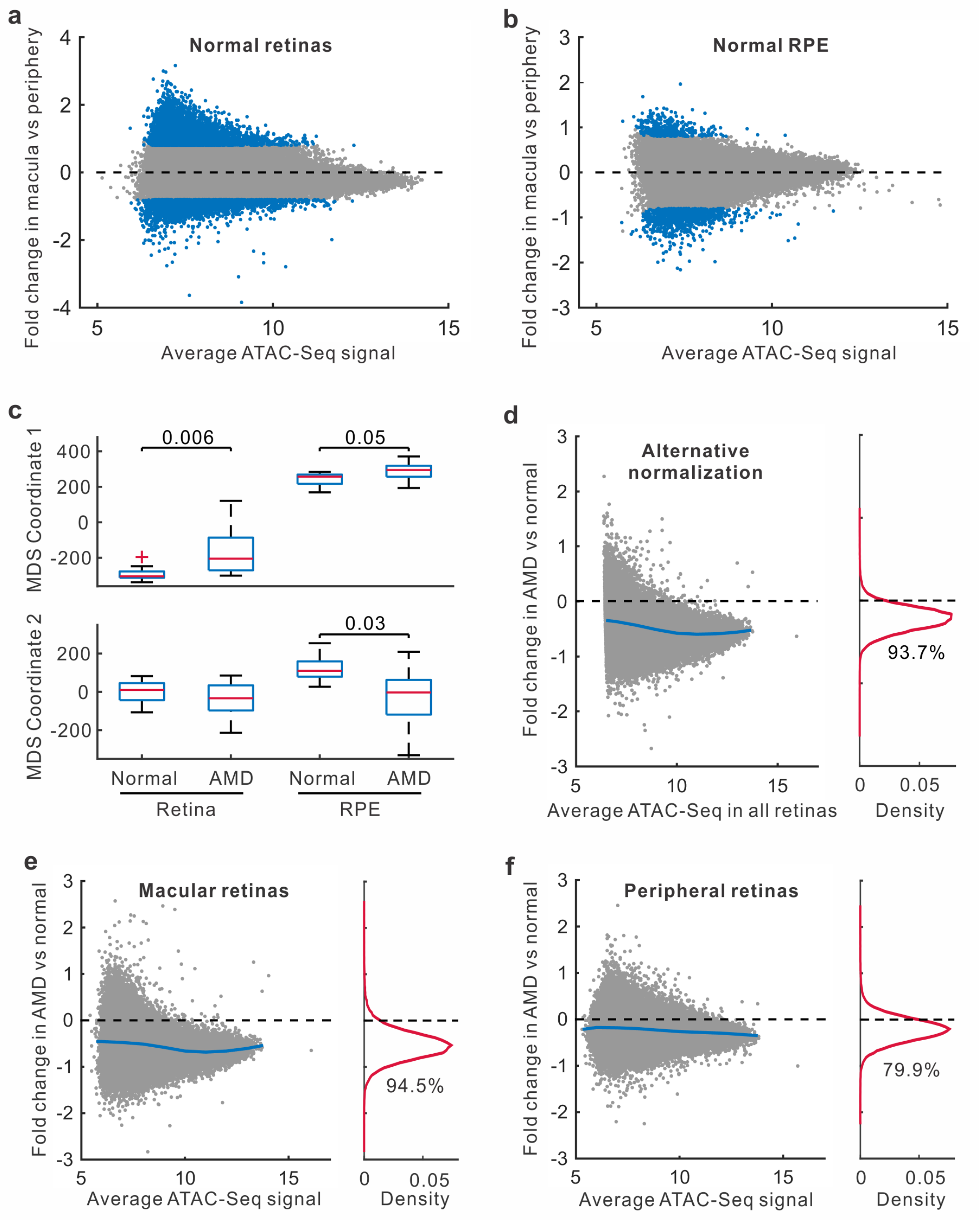

Supplementary Figure 2. Region-specific or disease specific changes in chromatin accessibility. **(a-b)** Changes of chromatin accessibility in macular vs. matched periphery regions of retina (n = 4) or RPE (n = 3) from healthy donors. Blue dots represent peaks with significantly differential accessibility. **(c)** Comparison of the difference in MDS coordinates between normal and AMD samples. **(d)** Changes of chromatin accessibility in all normal and AMD retinas using alternative normalization, in which we used the number of the all properly paired fragments as the library size. **(e-f)** Changes of chromatin accessibility in AMD relative to normal in retina samples, with macular and peripheral regions analyzed separately. Each dot represents one ATAC-Seq peak. Blue line indicates average fold changes of peaks. The percentage of peaks with reduced ATAC-Seq signal in AMD is shown under the density curve.

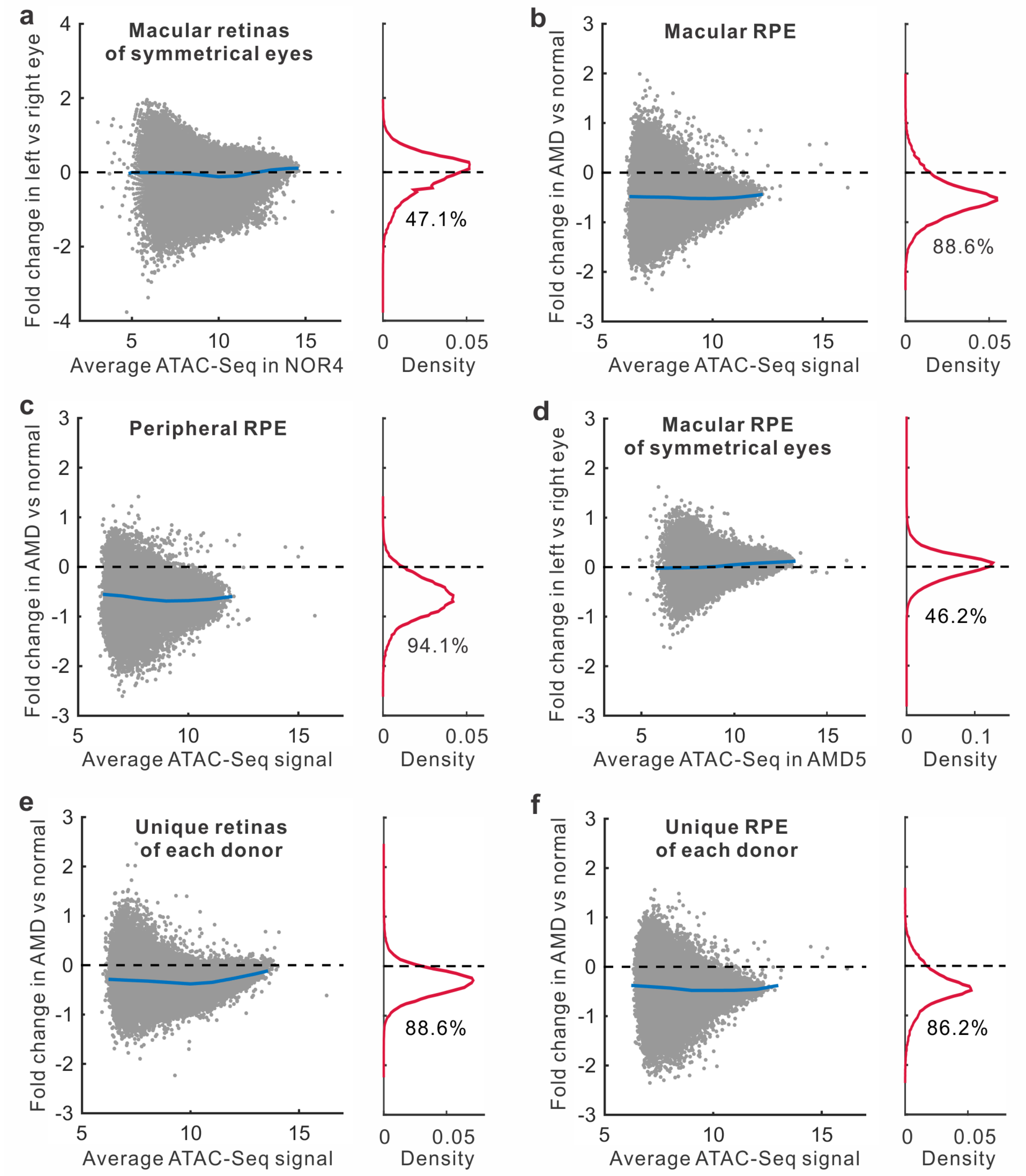

Supplementary Figure 3. Changes of chromatin accessibility in eyes from the same donor. **(a)** Changes of chromatin accessibility in macular retinas from the same donor whose eyes are at the same (symmetrical) stage of disease. Blue line represents the average change of ATAC-Seq peaks (gray dots). The percentage of peaks with the reduced accessibility is showed under the density curve in the right panel. **(b-c)** Changes of chromatin accessibility in AMD relative to normal in RPE samples from macular and peripheral regions, respectively. **(d)** Changes of chromatin accessibility in macular RPE from the same donor whose eyes are at the same stage of disease. **(e-f)** Changes of chromatin accessibility in AMD relative to normal samples. Only one sample was included from each donor.

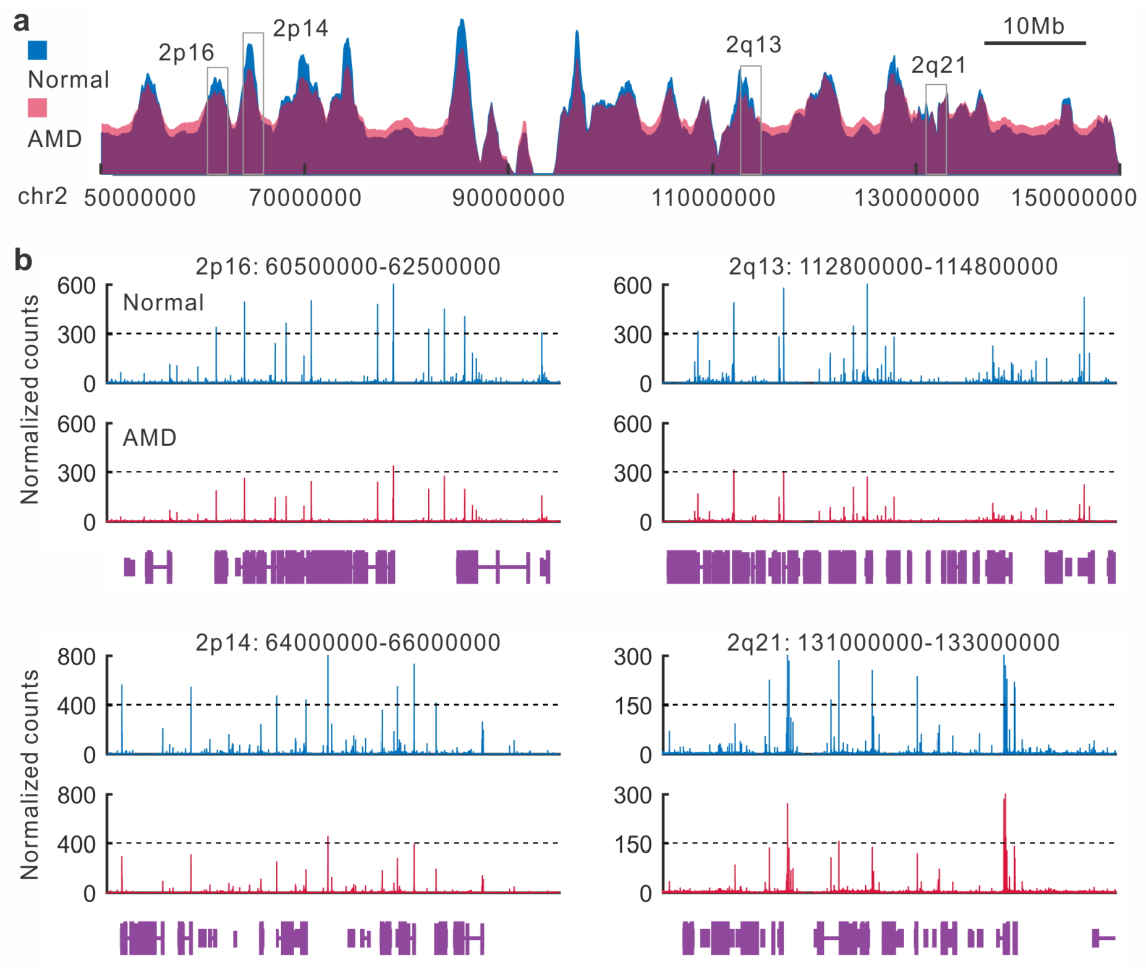

Supplementary Figure 4. Examples of large genomic domains with chromatin accessibility changes in the RPE. **(a)** Average ATAC-Seq intensity in AMD (red) and normal (blue) in a 100Mb genomic region. In this region, we can find many genomic domains (1-2Mb size), in which the ATAC-Seq signals in AMD samples are significantly lower than those in normal samples. **(b)** A few of these domains (2Mb each). The first three regions show the global reduction of ATAC-Seq signals in AMD, while the fourth example shows both reduced peaks in AMD and non-affected peaks.

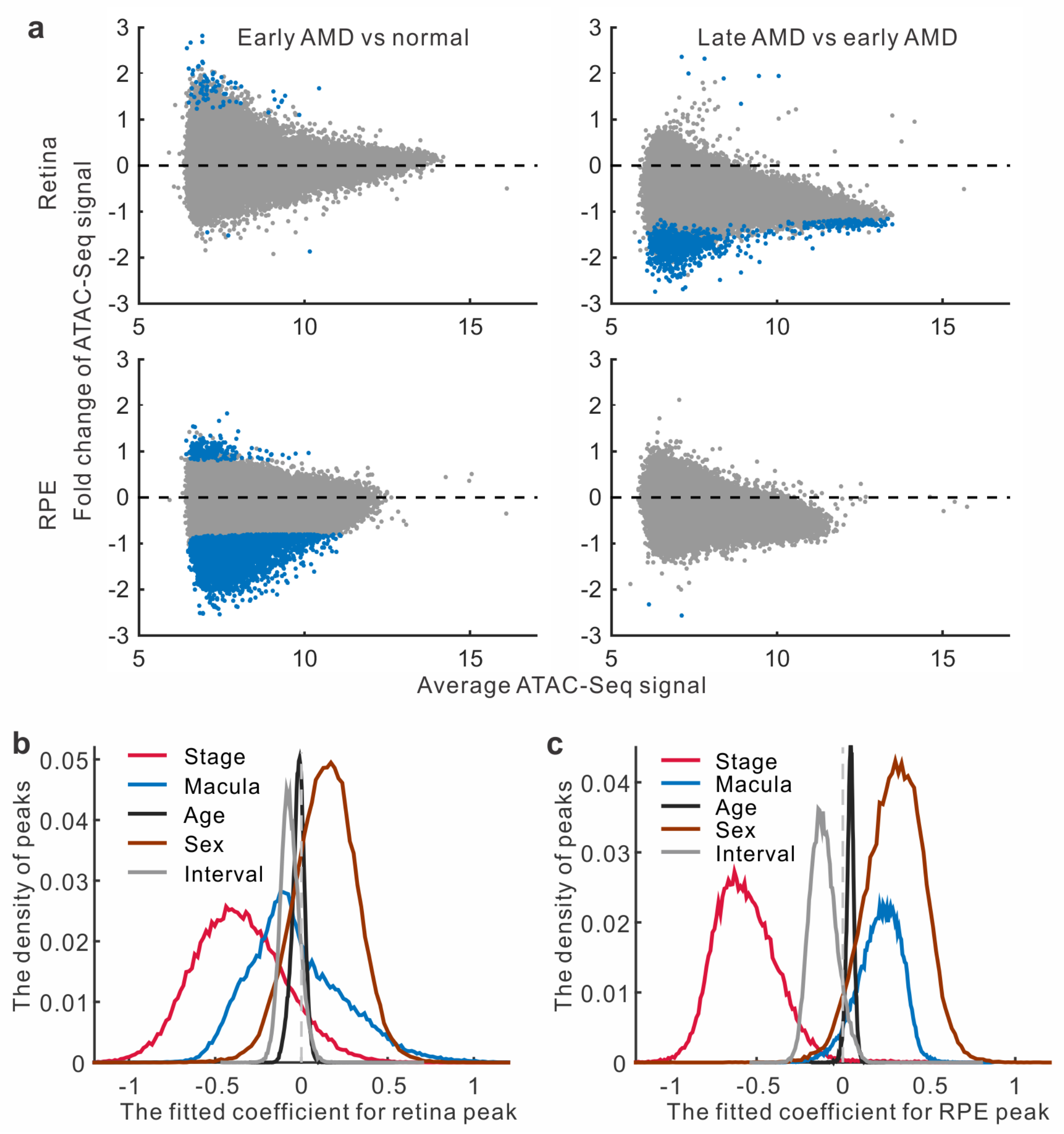

Supplementary Figure 5. Chromatin accessibility changes at different disease stages in the retina and RPE. **(a)** Changes of ATAC-Seq signal at different stages of AMD. The blue dots are peaks with significantly differential chromatin accessibility. **(b-c)** The distribution of the coefficients in the regression model of ATAC-Seq peaks from retina and RPE samples. For each ATAC-Seq peak, the coefficient of every variable was derived using the linear regression model.

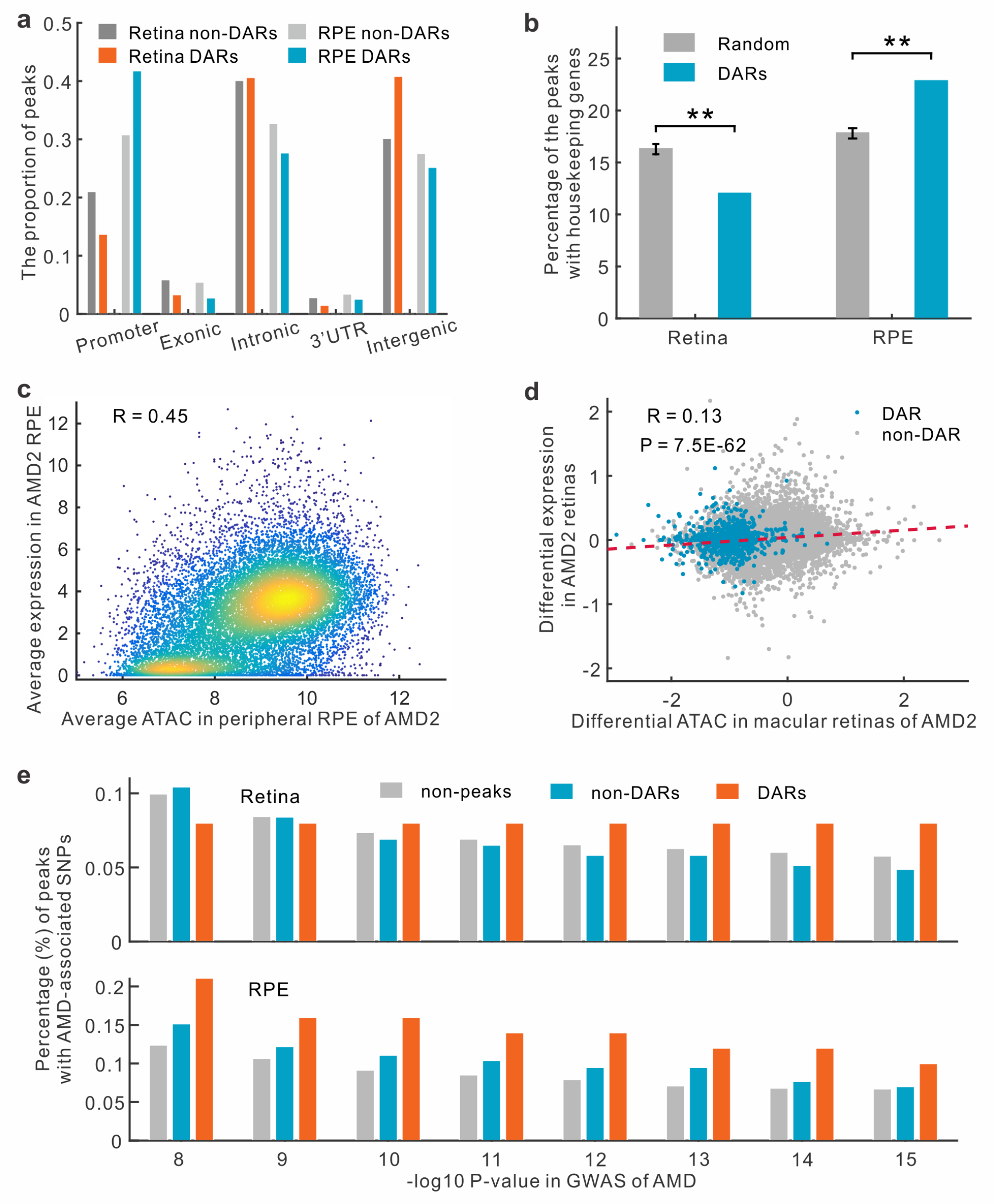

Supplementary Figure 6. Features and GWAS variants associated with differentially accessible regions (DARs). **(a)** The distribution of ATAC-Seq peaks at different genomic locations. **(b)** The percentage of DARs that are associated with housekeeping genes. The expectation from random set is from 5000 randomly selected ATAC-Seq peaks. Student's t-test was performed. Two asterisks indicate P-value < 0.01. **(c)** Comparison of chromatin accessibility and gene expression in the RPE of one AMD patient. Pearson's correlation coefficient (R) is shown. **(d)** Comparison of differential accessibility and differential expression in the retinas. The difference indicates the left eye (late-stage) relative to right eye (early-stage) of the AMD patient with asymmetric disease stages. **(e)** The percentage of ATAC-Seq peaks overlapped with significant GWAS SNPs. X-axis denotes the cutoff of GWAS significance. Non-peaks are the random regions outside the ATAC-Seq peaks. Non-DARs are the random ATAC-Seq peaks, which are not DARs.

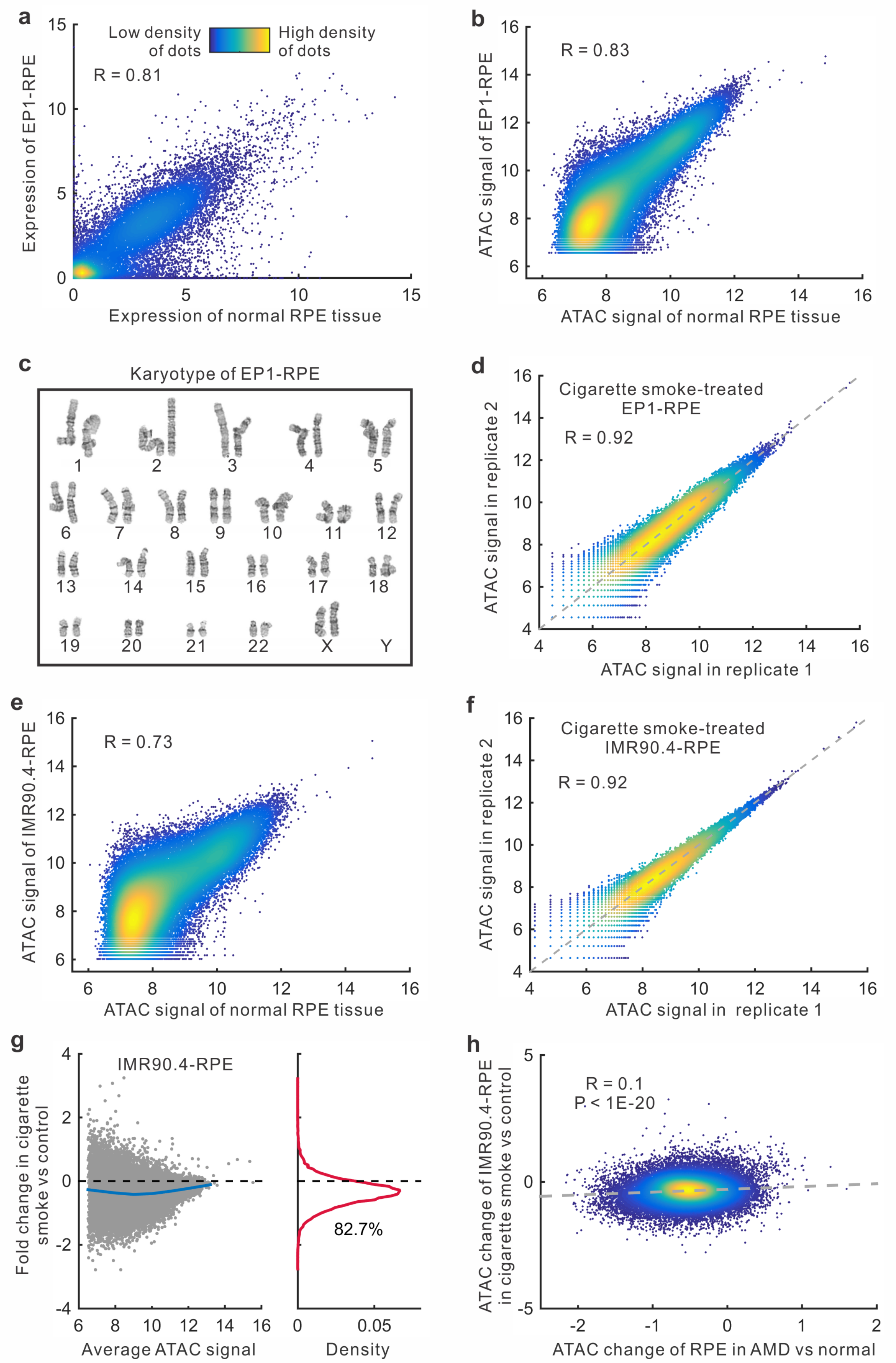
a
Low density of dots
High density of dots
R = 0.81
Expression of EP1-RPE
Expression of normal RPE tissue
b
R = 0.83
ATAC signal of EP1-RPE
ATAC signal of normal RPE tissue
c
Karyotype of EP1-RPE
1 2 3 4 5
6 7 8 9 10 11 12
13 14 15 16 17 18
19 20 21 22 X Y
d
Cigarette smoke-treated EP1-RPE
R = 0.92
ATAC signal in replicate 2
ATAC signal in replicate 1
e
R = 0.73
ATAC signal of IMR90.4-RPE
ATAC signal of normal RPE tissue
f
Cigarette smoke-treated IMR90.4-RPE
R = 0.92
ATAC signal in replicate 2
ATAC signal in replicate 1
g
IMR90.4-RPE
Fold change in cigarette smoke vs control
Average ATAC signal
82.7%
Density
h
R = 0.1
P. < 1E-20
ATAC change of IMR90.4-RPE in cigarette smoke vs control
ATAC change of RPE in AMD vs normal

Supplementary Figure 7. Expression profile and chromatin accessibility of iPSC-derived RPE cells. **(a)** Comparison of RNA-Seq gene expression profiles between normal RPE samples (n = 8) and iPSC-derived RPE cells (EP1-RPE, n = 3). **(b)** Comparison of ATAC-Seq profiles between normal RPE samples and iPSC-RPE. **(c)** Karyotype of iPSC-RPE cells. The cells demonstrated an apparently normal karyotype. **(d)** Comparison of ATAC-Seq profiles from two biological replicates of cigarette smoke-treated RPE cells. **(e-h)** ATAC-Seq profiles of cigarette smoke treatment of one additional cell line (IMR90.4). **(e)** Comparison of chromatin accessibility between iPSC-RPE cells and normal RPE tissue. **(f)** Comparison of chromatin accessibility in two biological replicates of iPSC-RPE cells. **(g)** Changes in chromatin accessibility of RPE cells after cigarette smoke treatment. The blue line represents the average change in ATAC-Seq peaks. The percentage of peaks with the reduced chromatin accessibility is showed under the density curve in the right panel. **(h)** Comparison of changes in chromatin accessibility between cigarette smoke-treated RPE cells and RPE from AMD donors.

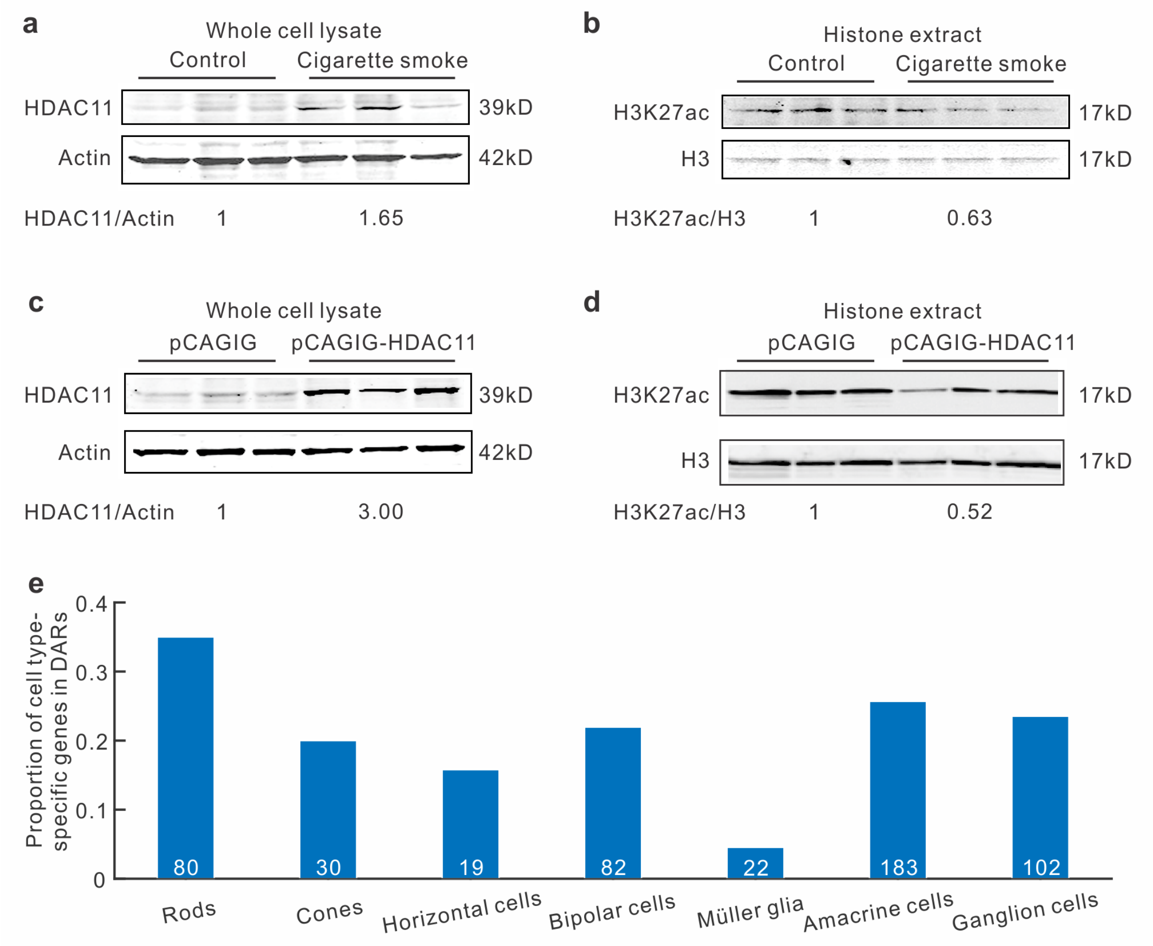

Supplementary Figure 8. *HDAC11* and H3K27ac levels change in iPSC-derived RPE cells treated with cigarette smoke. **(a)** Western blot of *HDAC11* following cigarette smoke treatment in human EP1-derived RPE cells. The number at the bottom of the image indicates the average abundance cross three lanes of each group. The average protein abundance in the control was normalized to 1. **(b)** Change of H3K27ac following cigarette smoke treatment. **(c)** *HDAC11* expression levels in cells overexpressing *Hdac11* (pCAGIG-HDAC11) compared with those in empty vector (pCAGIG). **(d)** Change of H3K27ac following *Hdac11* overexpression. The full Western blot images for (**a-d**) can be found in Supplementary Data 7. **(e)** The proportion of cell type-specific genes located in the retinal differentially accessible regions (DARs). The total number of cell type-specific genes in the study was shown at the bottom of the bar.

Supplementary Table 1. Donor characteristics

Variable	Norm (n = 5)	AMD (n = 5)	P value*
Gender (male)	2 (40%)	1 (20%)	0.41
Age (years)	84: 78 ~ 92	90: 85 ~ 95.5	0.24
No. of total eyes: No. of paired eyes#	8: 6	8: 6	0.99
Interval (hours)&	8.6	6	0.24

*Fisher's exact test and Student's t-test were performed respectively. #The paired eyes are eyes from the same donors. &Interval indicates the time from death to procurement of eyes.

Supplementary Table 2. Statistics of ATAC-Seq for retina and RPE samples

Sample*	Properly paired fragments (%)#	Qualified fragments&	Sample*	Properly paired fragments (%)#	Qualified fragments&
NOR1_Retina_MacR	95,198,063 (77.6)	56,151,841	AMD5_Retina_MacR_LA	36,530,166 (86.8)	22,515,903
NOR1_Retina_PerL	30,375,713 (84.0)	23,357,162	AMD5_Retina_PerR_LA	61,929,860 (83.5)	47,717,112
NOR1_Retina_PerR	66,360,580 (81.7)	41,210,030	NOR2_RPE_Mac	62,240,653 (86.6)	38,332,398
NOR2_Retina_Mac	47,063,121 (82.8)	31,460,883	NOR2_RPE_PerL	83,058,819 (83.6)	44,954,984
NOR2_Retina_Per	61,502,065 (85.6)	46,540,872	NOR2_RPE_PerR	54,504,714 (73.5)	30,156,496
NOR3_Retina_MacR	51,261,953 (84.2)	32,235,358	NOR3_RPE_PerR	47,401,144 (83.3)	35,286,709
NOR4_Retina_MacL	73,794,675 (77.9)	33,234,136	NOR4_RPE_MacL	80,538,584 (70.6)	55,001,840
NOR4_Retina_MacR	56,130,375 (79.4)	24,316,121	NOR4_RPE_MacR	87,406,027 (73.3)	47,954,810
NOR4_Retina_PerL	44,670,935 (79.4)	26,770,972	NOR4_RPE_PerL	95,699,676 (69.6)	65,998,781
NOR4_Retina_PerR	54,900,814 (76.3)	32,758,426	NOR4_RPE_PerR	95,508,108 (68.1)	60,201,309
NOR5_Retina_PerR	71,799,205 (90.5)	45,054,235	AMD1_RPE_MacL_LA	46,657,027 (85.3)	31,307,075
AMD1_Retina_MacL_LA	53,298,282 (81.3)	36,206,550	AMD1_RPE_MacR_LA	63,649,499 (81.0)	36,630,371
AMD1_Retina_MacR_LA	42,169,255 (82.0)	26,416,356	AMD1_RPE_PerR_LA	57,068,660 (80.9)	38,720,478
AMD1_Retina_PerR_LA	59,425,134 (85.1)	38,986,244	AMD2_RPE_MacL_LA	80,189,619 (75.9)	41,553,090
AMD2_Retina_MacL_LA	52,973,396 (76.4)	25,863,986	AMD2_RPE_PerL_LA	64,525,397 (67.8)	35,411,272
AMD2_Retina_MacR_EA	41,334,371 (75.6)	20,019,283	AMD2_RPE_PerR_EA	65,795,447 (65.1)	39,301,200
AMD2_Retina_PerL_LA	41,470,433 (77.4)	23,218,933	AMD3_RPE_MacR_EA	72,938,776 (78.6)	31,369,459
AMD2_Retina_PerR_EA	72,157,524 (69.2)	24,251,972	AMD3_RPE_PerR_EA	68,690,135 (75.0)	32,083,977
AMD3_Retina_PerR_EA	116,652,520 (86.1)	38,580,810	AMD4_RPE_MacR_EA	32,335,897 (83.3)	20,123,768
AMD4_Retina_MacR_EA	39,004,966 (83.0)	19,575,730	AMD5_RPE_MacL_LA	57,902,954 (81.4)	36,203,385
AMD4_Retina_PerR_EA_Rep1	51,899,554 (78.4)	38,903,055	AMD5_RPE_MacR_LA	42,040,435 (82.3)	32,761,740
AMD4_Retina_PerR_EA_Rep2	48,746,337 (79.7)	34,160,635	AMD5_RPE_PerR_LA	48,591,576 (82.6)	48,591,576
AMD5_Retina_MacL_LA	55,453,128 (85.5)	35,550,123			
AMD5_Retina_PerL_LA	45,593,306 (86.1)	32,117,341	Average	60,400,845 (78.5)	35,799,881

*The sample ID contains the following information: disease status of donors (NOR: normal; AMD: AMD), type of tissue (Retina vs RPE), region (Mac: macula; Per: periphery), eye index (R: right eye; L: left eye), and disease stage (EA: early stage; LA: late stage). For the sample of AMD4_Retina_PerR, there are two biological replicates (Rep1 and Rep2). # The percentage in the bracket represents properly paired fragments as a percentage of total raw fragments. & fragments with MAPQ score > 10 after removing fragments from mitochondrial DNA and chromosome Y, as well as duplicate fragments.

Supplementary Table 3. TF motifs associated with footprints within DARs from retinas and RPE

Transcription factor	Motif ID	FDR			Transcription factor	Motif ID	FDR		
		All	Proximal	Distal			All	Proximal	Distal
**From footprints within DARs of retinas**									
OTX2	M01719	7E-14	3E-3	3E-13	CRX	M00623	1E-6	0.04	1E-6
POU2F1	M00138	2E-6	0.09	4E-7	MEF2D	M00941	1E-4	8E-3	0.07
PAX6	M01391	5E-4	0.57	6E-6	FOXJ2	M00422	7E-4	8E-3	0.34
POU3F3	M01324	1E-3	0.09	0.01	TLX2	M01420	2E-3	0.23	3E-3
CDC5L	M00478	4E-3	0.01	0.63	RORA	M00157	8E-3	0.14	0.12
CUX1	M01344	9E-3	6E-3	0.99	RXRA	M01152	0.01	0.04	0.53
FOXO3	M01137	0.01	0.09	0.37	VAX2	M01327	0.01	0.57	2E-3
NKX3-1	M00451	0.01	0.03	0.68	PHOX2A	M01444	0.01	0.53	2E-3
MEIS1	M00420	0.02	0.09	0.51	POU6F1	M00465	0.02	0.32	0.06
ONECUT1, ONECUT2	M00639	0.03	0.25	0.11	IRX5	M01472	0.03	0.15	0.34
ZBTB16	M01075	0.04	0.08	0.90	GTF2IRD1	M01229	0.05	0.24	0.25
**From footprints within DARs of RPE**									
POU3F3	M01324	2E-4	0.20	8E-8	SOX5	M00042	2E-3	0.38	3E-9
SOX4	M01308	2E-3	0.02	0.07	CTNNB1	M03539	0.01	0.01	0.90
POU2F1	M00161	0.02	0.62	3E-6	PITX2	M01447	0.02	0.52	5E-5
CUX1	M00104	0.02	6E-4	0.99	NKX3-1	M00451	0.02	0.09	0.11
CEBPG	M00622	0.02	0.06	0.27	OTX2	M01719	0.02	0.46	3E-4
SATB1	M01723	0.02	0.64	1E-5	CDC5L	M00478	0.04	0.61	3E-3
TCF4	M02033	0.04	0.13	0.21					

Based on their positions relative to the gene body, all peaks were divided into two groups: proximal and distal. We calculated the FDRs for proximal and distal peaks, respectively.

Supplementary Table 4. The expression of histone deacetylases in RPE tissue

Gene	Probe ID	Normal (n = 96)	AMD (n = 30)	Log2 fold change	P value
HDAC1	12245	8.56	8.53	-0.04	0.54
HDAC2	40294	8.67	8.59	-0.08	0.30
HDAC3	29325	3.90	3.94	0.04	0.61
HDAC4	14394	5.49	5.61	0.12	0.20
HDAC5	3947	7.66	7.55	-0.11	0.23
HDAC6	25939	7.59	7.53	-0.06	0.39
HDAC7	9457	9.34	9.33	0.00	0.97
HDAC8	24646	4.15	4.20	0.04	0.61
HDAC9	33494	2.52	2.61	0.09	0.45
HDAC10	19616	3.53	3.24	-0.29	**0.02**
HDAC11	38006	4.17	4.41	0.24	**0.05**
SIRT1	41246	5.34	5.10	-0.24	**0.02**
SIRT2	5854	6.08	5.99	-0.09	0.24
SIRT3	8468	7.32	7.46	0.14	0.13
SIRT4	43993	3.13	3.13	-0.01	0.97
SIRT5	18987	6.31	6.18	-0.13	0.14
SIRT6	11898	5.17	5.19	0.02	0.76
SIRT7	26965	6.66	6.55	-0.11	0.25

Gene expression data were obtained from the microarray GSE29801. Both macular and peripheral RPE were included. Only dry AMD samples were used for this analysis.
