# Supplementary material for: ATAC-Seq analysis reveals a widespread decrease of chromatin accessibility in age-related macular degeneration
Source: Nat Commun. 2018 Apr 10;9:1364. doi: 10.1038/s41467-018-03856-y (PMC5893535; doi:10.1038/s41467-018-03856-y)
Supplement: Supplementary file 3 — Description of Additional Supplementary Files [file 41467_2018_3856_MOESM3_ESM.pdf]

Description of Additional Supplementary Files

File Name: Supplementary Data 1
Description: ATAC-Seq peaks from all retinal samples

File Name: Supplementary Data 2
Description: ATAC-Seq peaks from all RPE samples

File Name: Supplementary Data 3
Description: Differential ATAC-Seq peaks of macular vs. peripheral retinal samples from normal eyes

File Name: Supplementary Data 4
Description: Differential ATAC-Seq peaks of macular vs. peripheral RPE samples from normal eyes

File Name: Supplementary Data 5
Description: Description: Differentially accessible regions (DARs) in the retina identified by linear regression modeling of AMD and normal samples

File Name: Supplementary Data 6
Description: Differentially accessible regions (DARs) in the RPE identified by linear regression modeling of AMD and normal samples

File Name: Supplementary Data 7
Description: Full Western blot images for HDAC11 and H3K27ac in CSE treatment and HDAC11 overexpression cells.
