# Supplementary figures and images for: ATAC-Seq analysis reveals a widespread decrease of chromatin accessibility in age-related macular degeneration
Source: Nat Commun. 2018 Apr 10;9:1364. doi: 10.1038/s41467-018-03856-y (PMC5893535; doi:10.1038/s41467-018-03856-y)

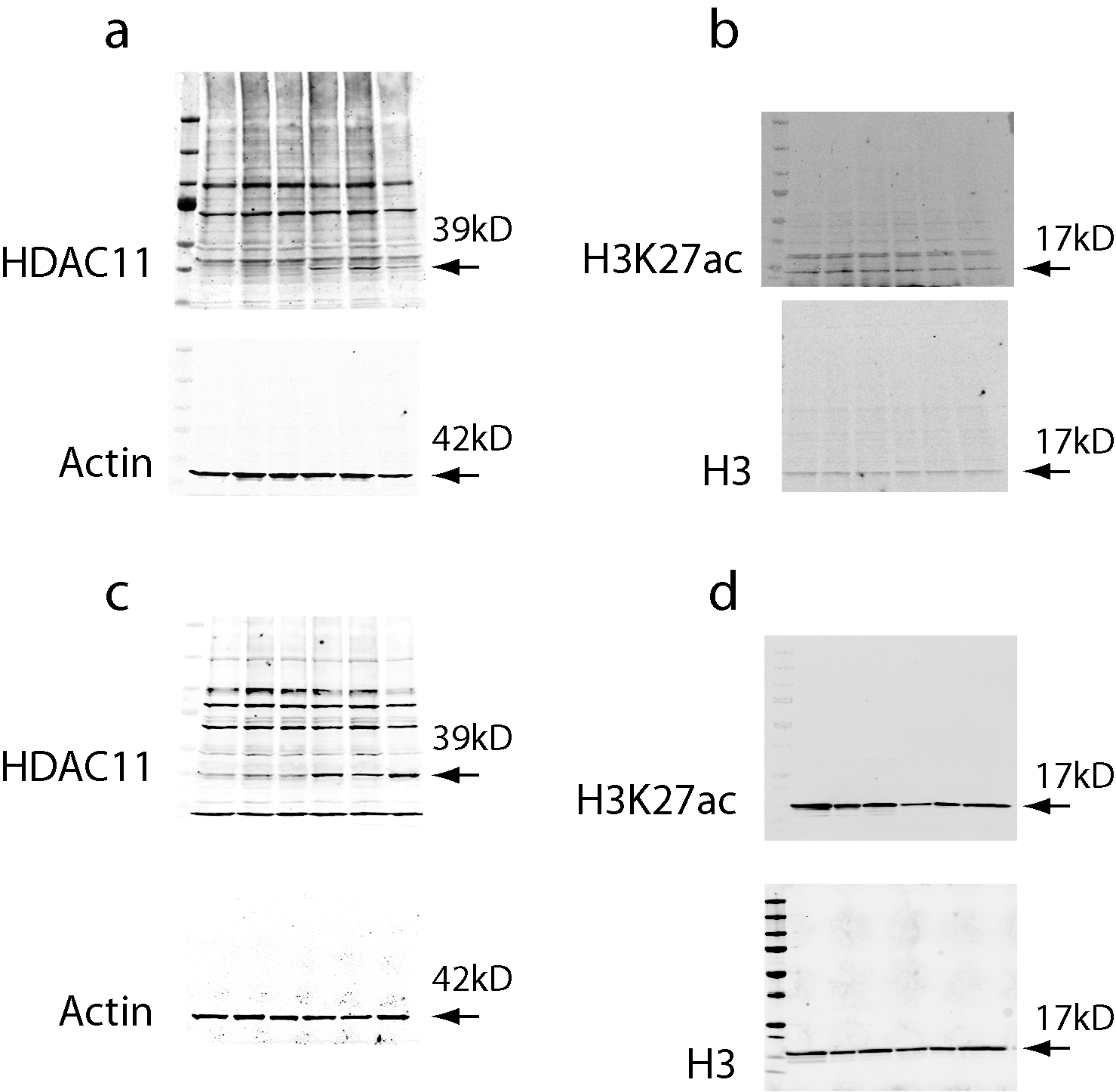

Supplement: Supplementary file 10 — Supplementary Data 7 [file 41467_2018_3856_MOESM10_ESM.tif]
